# Early clinical outcomes of bipolar hemiarthroplasty for femoral neck fractures in elderly patients using the OCM approach: a retrospective study

**DOI:** 10.3389/fsurg.2024.1396717

**Published:** 2024-07-05

**Authors:** Hongming Zheng, Danhui Kong, Shuangjun He, Boyi Jiang, Dongbo Zhu, Shuhua Wu, Yaowei Wang, Lijian Zhou, Yan Xia

**Affiliations:** Department of Orthopedic Surgery, Affiliated Danyang Hospital of Nantong University, The People’s Hospital of Danyang, Danyang, China

**Keywords:** femoral neck fractures, elderly patients, bipolar hemiarthroplasty, OCM approach, early clinical outcomes, aged 75 and above

## Abstract

**Objective:**

This study aims to assess the early clinical outcomes of bipolar hemiarthroplasty for treating femoral neck fractures in elderly patients aged 75 and above using the Orthopädische Chirurgie München (OCM) approach.

**Methods:**

A retrospective analysis was conducted on a cohort of 95 elderly patients who underwent bipolar hemiarthroplasty for Garden Type III and IV femoral neck fractures between January 2020 and December 2022. The participants were categorized into two groups according to the surgical approach used: the OCM approach and the posterior-lateral approach (PLA). The average follow-up duration was 11.20 ± 2.80 months for the OCM group and 11.12 ± 2.95 months for the PLA group, with both groups ranging from 6 to 18 months. Clinical outcomes assessed included surgical duration, incision length, postoperative hospital stay, time to ambulation, hemoglobin levels, serum creatine kinase (CK) levels, C-reactive protein (CRP) levels, pain (assessed using the Visual Analogue Scale, VAS), and functional recovery (evaluated through Harris hip scores). Additionally, complications such as intraoperative and postoperative fractures, deep vein thrombosis, wound infection, nerve injury, postoperative dislocation, leg length discrepancy, and Trendelenburg gait were monitored.

**Results:**

There was no significant difference in the surgical duration between the OCM and PLA groups. However, the OCM group exhibited shorter incision lengths, reduced postoperative hospital stays, and earlier ambulation times compared to the PLA group. Significantly lower intraoperative blood loss, smaller decreases in hemoglobin levels on postoperative days 1 and 3, lesser hidden blood loss, and decreased levels of CK and CRP were observed in the OCM group. Pain levels, measured by VAS scores, were lower, and Harris hip scores, indicating functional recovery, were higher at 2 and 6 weeks postoperatively in the OCM group than in the PLA group. The incidence of complications, such as periprosthetic fractures, intramuscular venous thrombosis, hip dislocations, Trendelenburg gait, and leg length discrepancies, showed no significant differences between the groups.

**Conclusion:**

The OCM approach for bipolar hemiarthroplasty in patients aged 75 and above with femoral neck fractures offers significant early clinical benefits over the traditional PLA, including faster recovery, reduced postoperative pain, and enhanced early functional recovery.

## 1 Introduction

With the continuing trend of an aging population, the incidence of hip fractures in the elderly is on the rise. It is estimated that by 2050, this number will increase to over five million globally (1). Among hip fractures, femoral neck fractures (FNFs) are the most common, accounting for approximately 53% of all hip fractures (2). Osteoporosis, prevalent in the elderly, makes even low-energy trauma sufficient to cause FNF, leading to decreased mobility and potentially death, thus imposing significant burdens on society and the economy (3–5). In China, risk factors for femoral neck fractures include advanced age, female gender, low body mass index (BMI), and osteoporosis (6).

Currently, hemiarthroplasty has become the primary treatment method for displaced femoral neck fractures in elderly patients over 75 years old, especially those who are frail and have lower physical demands, as it offers faster and satisfactory functional recovery (7). With the gradual adoption of the concept of fast-track recovery, various minimally invasive approaches to hip arthroplasty have emerged. Among them, the minimally invasive anterolateral approach (Orthopädische Chirurgie, München, OCM) was first introduced by Röttinger in 2004 (8). This approach, which navigates through the interval between the gluteus medius and tensor fasciae latae muscles without necessitating muscle cutting during surgery, offers several advantages, including minimal trauma, reduced pain, and accelerated recovery.

Multiple studies have documented favorable outcomes with the OCM approach, highlighting its effectiveness and safety. Müller et al. (9), Hansen et al. (10), and Shigemura et al. (11) have all noted that the OCM approach preserves muscle integrity, reduce damage, and effectively prepares the femur, leading to improved clinical outcomes. Despite similar operative times and radiological outcomes compared to traditional approaches, these minimally invasive techniques have shown superior clinical results, such as higher postoperative Harris hip scores and reduced muscle and tendon damage. However, others reported higher complications rate and a relatively slow learning curve (12, 13).

Although the benefits of using bipolar prostheses with the OCM approach for hemiarthroplasty in elderly patients with femoral neck fractures are not fully established, this study seeks to explore its potential advantages and early effectiveness. We conducted a retrospective analysis comparing various clinical indicators such as operative time, perioperative and hidden blood loss, soft tissue damage, pain levels, and postoperative functional recovery between the OCM and posterior-lateral approach (PLA). Our goal is to provide a more objective foundation for choosing hemiarthroplasty procedures.

## 2 Materials and methods

### 2.1 Study design

This was a retrospective analysis of patients with femoral neck fractures. This study strictly follows the guidelines of the ethical censorship of the People's Hospital of Danyang and has been also approved by the Ethics Committee of the People's Hospital of Danyang.

### 2.2 Inclusion and exclusion criteria

Inclusion criteria were the following: (1) Patients aged 75 years or older; (2) Patients with unilateral fresh femoral neck fractures (Garden III and IV); (3) Patients with a BMI under 30 kg/m^2^; (4) Patients capable of ambulation without assistive devices prior to the injury; (5) Patients free from active infections; (6) Patients with an American Society of Anesthesiologists (ASA) classification of I–III.

Exclusion criteria were the following: (1) Patients with sciatic nerve injury; (2) Patients with pathological fractures, femoral head necrosis, or arthritis of the affected hip joint; (3) Patients with a history of trauma and fractures in other parts of the body; (4) Patients with severe dysfunction of vital organs (heart, lungs, liver, kidneys) or blood disorders.

### 2.3 Patients

Between January 2020 and December 2022, a total of 95 patients who underwent bipolar hemiarthroplasty for femoral neck fractures and met the inclusion criteria were recruited. Patients were divided into two groups based on the surgical approach used: the OCM approach group (45 cases) and the posterior-lateral approach group (PLA group) (50 cases). Patient demographics, detailed in Table 1, include age, gender, BMI, Garden fracture classification, time from fracture to surgery, and the prevalence of comorbid conditions, with no significant differences observed between the groups.

**Table 1:** Patient data and demographics in the PLA and OCM groups.

	PLA group (*n* = 50)	OCM group (*n* = 45)	*t*/*χ*^2^ value	*P*-value
Age (years)	83.12 ± 5.189	82.24 ± 4.709	*t* = 0.858	0.393
Male/Female	21/29	19/26	χ^2^ = 0.000	0.983
BMI (kg/m^2^)	21.16 ± 2.698	21.27 ± 2.444	*t* = 0.201	0.841
Garden type III/IV	27/23	21/24	χ^2^ = 0.510	0.475
Time to surgery (h)	36.32 ± 4.240	35.53 ± 4.414	*t* = 0.886	0.378
Hypertension	32	28	χ^2^ = 0.032	0.858
Cardiopathy	8	6	χ^2^ = 0.134	0.714
Diabetes	16	14	χ^2^ = 0.009	0.926
Cerebrovascular accident	9	7	χ^2^ = 0.101	0.751
Chronic bronchitis	10	8	χ^2^ = 0.076	0.783

### 2.4 Surgical procedure

The surgeries were performed by two senior orthopedic surgeons within a unified treatment team, each with over ten years of experience specializing in their respective approach, well beyond the learning curve. Patients received either spinal or general anesthesia and were positioned in the lateral decubitus position with foam padding at the sacrum and pubic symphysis for stabilization. Intravenous tranexamic acid was administered to minimize bleeding.

#### 2.4.1 OCM approach

**Figure 1:**
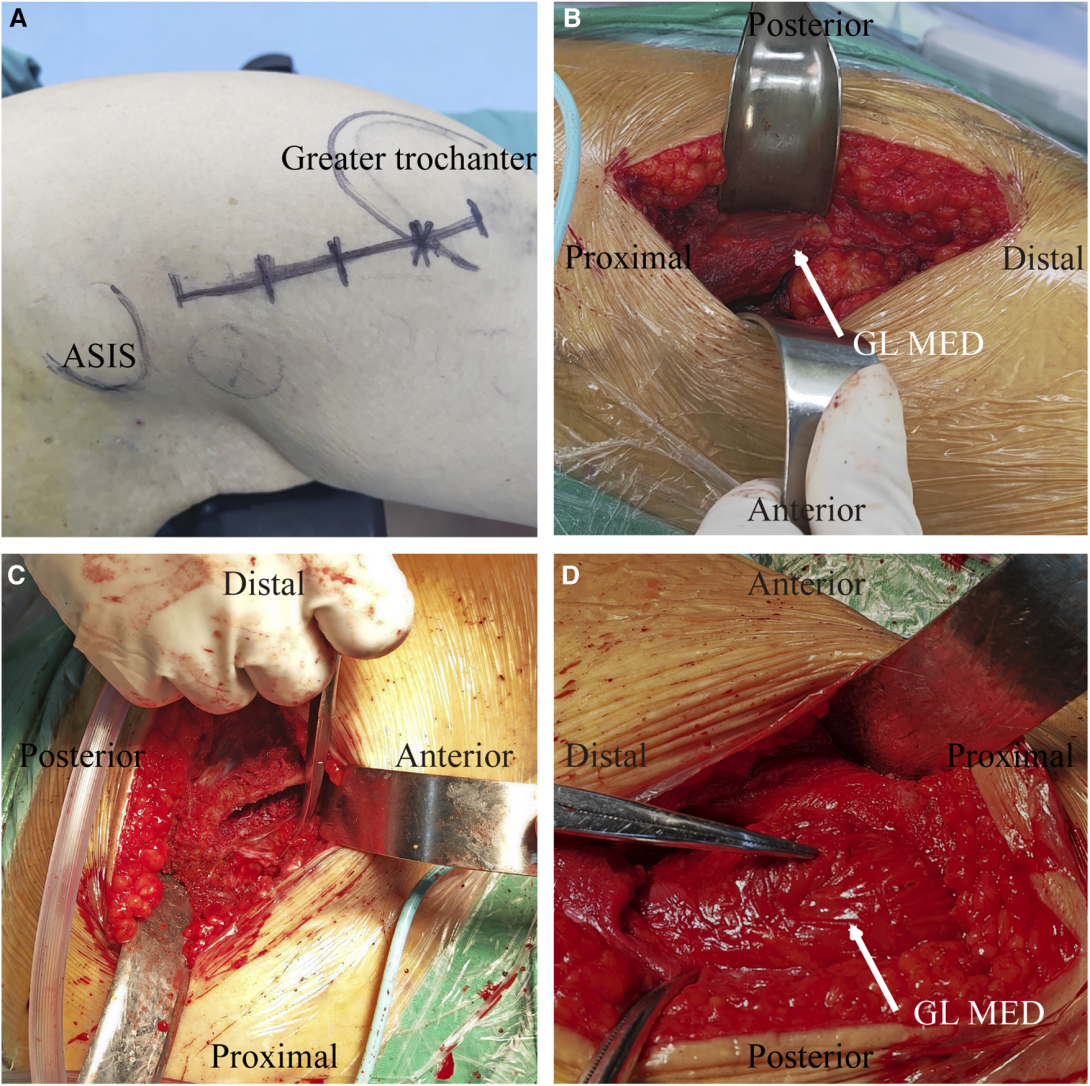
Operative photograph of OCM approach. (**A**) A 7–9 cm incision was made from the anterior aspect of the greater trochanter to the anterior superior iliac spine; (**B**) The interval between the tensor fascia and gluteus medius (white arrow) is exposed; (**C**) Any residual capsule at the margin of the lateral femoral neck is removed to expose the medullary cavity of the proximal femur; (**D**) Only minor gluteus medius contusion (white arrow) occurred after surgery. GL MED, gluteus medius.

**Figure 2:**
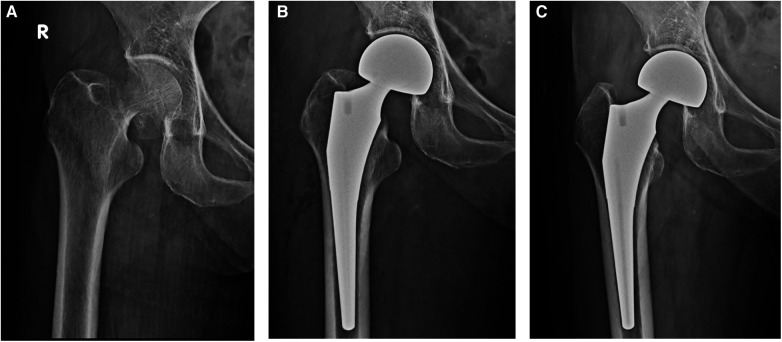
(**A**) Preoperative x-ray. Garden Type IV intracapsular femoral neck fracture of an 83 years old female patient. (**B**) Postoperative x-ray. Cementless metaphyseal porous coated stem with bipolar head were implanted using OCM approach. (**C**) 3 months Postoperative x-ray.

The patient is placed in a lateral decubitus position on a split operating table with the affected side up. The patient is positioned slightly forward so that the contralateral leg extends over the front section of the table's lower part. The back section is removed to allow the operative leg to be lowered during the procedure, facilitating adduction, extension, and external rotation of the femur for easier access during reaming and femoral stem insertion. A 7–9 cm incision was made from the anterior aspect of the greater trochanter to the anterior superior iliac spine, entering through the interval between the gluteus medius and tensor fasciae latae. Retractors are placed both above and below the femoral neck. An “H” shaped capsulotomy is performed from the intertrochanteric line to the acetabulum, fully exposing the femoral head-neck junction and the entire neck. The femoral neck osteotomy is carried out with the neck kept parallel to the floor, and the head is removed from the acetabulum using either a corkscrew. Then the leg is positioned in a sterile drape behind the patient in adduction, extension, and external rotation, allowing clear access to the medullary canal (Figures 1, 2). If necessary, the joint capsule around the greater trochanter and the conjoint tendons of the obturator internus and gemelli muscles are released to expose the proximal femur. The canal is prepared with reamers and rasps, and the femoral stem is inserted. Following trials, a bipolar head with the suitable neck length is selected and installed. The hip is then repositioned and its stability verified. The capsular sleeves are approximated, the fascia is securely sutured, and the subcutaneous tissue and skin are closed.

#### 2.4.2 Posterior-lateral approach

The patient was positioned in a lateral decubitus position. The surgical incision commenced 10 cm below the posterior superior iliac spine and extended to the posterior edge of the greater trochanter. The incision length was between 10 and 12 cm, with the division of the deep fascia aligned with the skin incision. Blunt dissection was used to separate the fibers of the gluteus maximus, revealing the greater trochanter. The distal fibers and external rotators were exposed and released. The muscles were retracted medially to expose and longitudinally split the joint capsule from the distal to the proximal end along the femoral neck, detaching the distal portion of the capsule from the femur and the edge of the acetabulum. Standard posterior approach techniques were employed for the femoral neck osteotomy, followed by posterior dislocation of the hip and prosthesis implantation. The joint capsule was reconstructed as effectively as possible, and the external rotator cuff was sutured and secured to the greater trochanter.

Both groups received bipolar and wedge-shaped prostheses with uncemented fixation. Prosthesis positioning was verified using fluoroscopy.

### 2.5 Postoperative care

Upon regaining consciousness post-anesthesia, patients were encouraged to begin ankle pump exercises in bed. In the OCM group, there were no post-surgery limb positioning restrictions. In contrast, the PLA group avoided lower limb adduction, placing a triangular cushion between the lower limbs to prevent internal rotation of the hip joint. Furthermore, hip flexion was restricted to 90°. From the first day post-surgery, patients in both groups were allowed to initiate weight-bearing activities as tolerated and, by the second day, were encouraged to get out of bed with assistance, walk on flat surfaces, and climb stairs with a cane if their condition permitted. Discharge criteria encompassed the ability to independently get up and use the restroom and safe walking with assistive devices.

Infection prevention included administering routine intravenous cefuroxime 24 h after surgery. Our standard postoperative pain management protocol involved on-demand nonsteroidal anti-inflammatory drugs, supplemented with opioids as needed. Anticoagulation management utilized low molecular weight heparin sodium (5,000 U, once daily). Except for those with contraindications such as recent deep vein thrombosis, bleeding tendencies, or deep vein thrombophlebitis, patients received bilateral lower limb intermittent pneumatic compression therapy for 45 min twice daily to prevent venous thrombosis in the legs. Doppler ultrasounds of the lower extremity veins were performed when any symptom or sign suspicious of venous thromboembolism was observed, to detect deep vein thrombosis and pulmonary embolism. Suspicious symptoms and signs included swelling or redness in the foot and ankle, pain or tenderness in the leg, Homans’ sign, and dyspnea. Upon discharge, patients were prescribed oral rivaroxaban (5 mg, once daily). Blood transfusions were considered for patients with hemoglobin levels below 85 g/L or those exhibiting clinical symptoms like tachycardia, hypotension, or anemia.

### 2.6 Evaluation of clinical outcomes

Intraoperative blood loss (IBL) was calculated by summing the fluid volume in the suction canister with the increase in gauze weight, then subtracting the irrigating fluid volume. Visible blood loss (VBL) was defined as IBL plus drainage output, while hidden blood loss (HBL) was determined as total blood loss (TBL) minus VBL (14). TBL (ml) was calculated using the equation (15): TBL= Preoperative total blood volume (PBV) × [Hctpre − (Hct1 + Hct3) / 2] + transfusion volume, where Hct_pre is the preoperative hematocrit, and Hct_1 and Hct_3 are hematocrit values on the first and third postoperative days, respectively. PBV (ml) was determined by: $\mathrm{PBV}=[k1\times \mathrm{height}(m{)}^{3}+k2\times \mathrm{weight}(\mathrm{kg})+k3\;]\times 1000$, with gender-specific k values (for males, k1 = 0.3669, k2 = 0.03219, k3 = 0.6041; for females, k1 = 0.3561, k2 = 0.03308, k3 = 0.1833) (16).

Serum creatine kinase (CK) and C-reactive protein (CRP) levels were measured preoperatively and on days 1, 3, and 7 post-surgery to assess inflammation or tissue damage (17). Pain intensity was evaluated at 12, 48, and 72 h postoperatively using the Visual Analogue Scale (VAS), and hip joint function was assessed at 2, 6, and 12 weeks postoperatively with the Harris Hip Score (HHS). Leg length discrepancy and Trendelenburg gait were also evaluated at 12 weeks post-surgery.

### 2.7 Statistical analysis

Statistical analysis was performed using GraphPad Prism 6 (GraphPad, CA). Continuous data, presented as mean ± standard deviation, were compared between groups using Student's *t*-test or repeated measures analysis of variance for within-group comparisons, following normality testing. Categorical variables, shown as frequencies and percentages, were analyzed with the chi-squared or Fisher's exact test as appropriate. A *p*-value of less than 0.05 was considered statistically significant.

## 3 Results

The average follow-up duration was similar between groups, at 11.20 ± 2.80 months for the OCM group and 11.12 ± 2.95 months for the PLA group, both ranging from 6 to 18 months. This similarity in observation periods underscores the comparability of the two groups.

Surgical durations were equivalent; however, the OCM group exhibited benefits such as shorter incisions and quicker recovery times in terms of ambulation and hospital stays, suggesting a more efficient and less invasive approach (refer to Table 2).

**Table 2:** General surgical data comparison between PLA and OCM groups.

	PLA group	OCM group	*t*-value	*P*-value
Surgery time (min)	63.48 ± 3.541	68.60 ± 7.533	1.784	0.078
Incision length (cm)	10.04 ± 0.3476	8.911 ± 0.2878	17.13	<0.001*
Postoperative hospital stay (days)	5.16 ± 0.8929	4.733 ± 0.9529	2.093	0.039
Time to ambulation (h)	48.60 ± 12.05	25.40 ± 4.845	12.06	<0.001*

* Indicates statistical significance.

There were no significant differences in preoperative hemoglobin or blood volume levels between the groups. Nonetheless, the OCM group achieved superior perioperative blood management, evidenced by significantly lower intraoperative and postoperative blood loss, although transfusion rates did not differ significantly (refer to Table 3).

**Table 3:** Comparison of perioperative blood loss between PLA and OCM groups.

	PLA group	OCM group	*t*/χ^2^ value	*P*-value
IBL (ml)	131.5 ± 35.9	104.0 ± 15.08	*t* = 4.775	<0.001*
Preoperative Hb (g/L)	116.7 ± 11.55	115.8 ± 10.37	*t* = 0.379	0.705
*Δ*Hb1 (g/L)	18.48 ± 4.929	14.78 ± 4.527	*t* = 3.799	0.003*
*Δ*Hb3 (g/L)	24.40 ± 6.243	20.80 ± 6.144	*t* = 2.827	0.006*
PVB (ml)	2,897 ± 254.5	2,933 ± 254.1	*t* = 0.683	0.497
HBL (ml)	280.3 ± 91.70	203.1 ± 51.08	*t* = 4.992	<0.001*
Need for transfusion [cases (%)]	8/50 (16)	4/45 (9)	χ^2^ = 1.085	0.298

IBL, intraoperative blood loss; PBV, preoperative total blood volume; HBL, hidden blood lossthe; Hb, hemoglobin; ΔHb1 and ΔHb3, reduction in Hb levels on the 1st and 3rd postoperative days respectively.

* Indicates statistical significance.

Regarding serological markers, there were no preoperative differences; however, postoperative analyses revealed significantly lower levels of CK and CRP in the OCM group, indicating reduced muscle damage and less inflammatory response (refer to Table 4). Additionally, pain management was markedly more effective in the OCM group, as demonstrated by lower VAS scores at 12, 24, and 72 h post-surgery (refer to Table 5).

**Table 4:** Comparison of serological markers between PLA and OCM groups.

	PLA group	OCM group	*t*-value	*P*-value
CK (IU/L)				
Preoperative	73.04 ± 26.17	65.27 ± 29.40	1.364	0.176
1 day post-surgery	332.4 ± 94.86	258.8 ± 69.77	4.269	<0.001*
3 day post-surgery	216.8 ± 65.28	169.3 ± 50.92	4.048	<0.001*
CRP (mg/L)				
Preoperative	29.90 ± 10.40	27.93 ± 7.709	1.037	0.302
1 day post-surgery	111.0 ± 14.67	99.87 ± 25.55	2.642	0.010*
3 day post-surgery	93.52 ± 20.14	74.49 ± 20.58	4.551	<0.001*

* Indicates statistical significance.

**Table 5:** Comparison of VAS scores between PLA and OCM groups at various postoperative intervals.

	PLA group	OCM group	*t*-value	*P*-value
Preoperative	5.120 ± 0.9179	5.289 ± 0.7869	0.958	0.341
12 h post-op	4.260 ± 0.6642	3.844 ± 0.706	2.956	0.004*
24 h post-op	2.860 ± 0.7001	2.378 ± 0.833	3.063	0.003*
72 h post-op	1.800 ± 0.7825	1.200 ± 0.405	4.617	<0.001*

VAS, visual analog scale, used to measure pain intensity.

* Indicates statistical significance.

Functional outcomes, as measured by Harris scores, were initially better in the OCM group at 2 and 6 weeks postoperatively. However, by 6 months, there were no significant differences, suggesting similar long-term outcomes (refer to Table 6).

**Table 6:** Comparison of Harris scores between PLA and OCM groups at various postoperative intervals.

	PLA group	OCM group	*t*-value	*P*-value
2 weeks	52.70 ± 909.2	63.02 ± 5.825	6.105	<0.001*
6 weeks	72.22 ± 2.909	74.29 ± 5.558	2.305	0.023*
6 months	94.02 ± 2.511	95.00 ± 2.739	1.820	0.072

* Indicates statistical significance.

During postoperative monitoring, no cases of deep vein thrombosis, wound infections, or nerve injuries were observed in either group, and there were no deaths during the postoperative period. All prosthetic placements were deemed satisfactory. Despite some complications such as periprosthetic fractures, asymptomatic intramuscular venous thrombosis, and hip joint dislocations, these were effectively managed with appropriate medical interventions: periprosthetic fractures were treated with cerclage wiring; patients with asymptomatic intramuscular venous thrombosis received standard anticoagulation therapy; and hip dislocations were corrected through manual manipulation and skin traction. At the three-month follow-up, although a few patients exhibited a Trendelenburg gait and slight leg length discrepancies (all under 10 mm), statistical analysis revealed no significant differences in the incidence of complications between the two groups (for detailed data, refer to Table 7), indicating that both surgical methods are comparable in terms of safety.

**Table 7:** Incidence of postoperative complications in PLA and OCM groups.

	PLA group (*n* = 50)	OCM group (*n* = 45)	χ^2^ value	*P*-value
Periprosthetic fractures	2 (4%)	1 (2.2%)		>0.999
Intramuscular venous thrombosis	4 (8%)	2 (4.44%)		0.680
Posterior hip joint dislocation	1 (2%)	0 (0%)		>0.999
Trendelenburg gait at 3 months	7 (14%)	2 (4.44%)		0.164
Leg length discrepancy	27 (54%)	21 (46.67%)	0.369	0.544

## 4 Discussion

Our study highlights the distinct advantages of the OCM approach over the posterior-lateral approach in performing bipolar hemiarthroplasty for elderly patients. It emphasizes significantly enhanced early functional recovery and reduced postoperative pain. This selection is pivotal for patients aged 75 and older due to their specific physiological challenges and the urgent need for rapid recovery to maintain independence.

Recent literature supports the widespread adoption of various minimally invasive techniques in total hip arthroplasty, with the OCM approach noted for its reduced surgical trauma, blood loss, and quicker recovery (9–11, 18–20). However, the OCM approach requires a high level of surgical expertise and specific instruments, which could increase the complexity and cost of the procedure (18). Initially, this method may lead to longer surgical durations as surgeons climb the learning curve, potentially increasing the risk of complications such as fractures (21, 22).

In our study, no significant differences in surgical times were observed between the OCM and PLA groups, indicating that adopting the OCM approach does not extend the duration of surgery. Although initial surgeries in the OCM group were longer, this discrepancy was linked to the surgeons' growing proficiency. We recommend that surgeons perform at least 15 hip arthroplasties using the OCM approach to ensure adequate mastery and safety, thus reducing surgical risks and enhancing outcomes.

The OCM group experienced lower intraoperative and postoperative blood loss, crucial for decreasing surgery-related infection risks—significant blood loss during surgery is a well-known risk factor for increased infection rates (23). Additionally, the OCM group showed lower postoperative levels of CK and CRP, alongside better Harris hip scores and VAS scores, indicating lesser surgical trauma and inflammation, which facilitated pain reduction. These advantages allowed for earlier mobilization and shorter hospital stays.

At 6 weeks postoperatively, the differences in outcomes between the OCM and PLA groups, although statistically significant, were relatively small, suggesting limited clinical impact. The lower Harris hip scores in the PLA group can be attributed to greater surgical trauma, increased postoperative pain, higher blood and inflammatory responses, restricted activities, and difficulties in rehabilitation. These factors collectively limit early functional recovery in the PLA group. While the benefits in Harris hip scores diminished by the six-month follow-up, suggesting that long-term recovery may be more influenced by patient-specific factors and rehabilitative support, the early benefits of the OCM approach remain clear.

We did not observe serious complications such as deep vein thrombosis and nerve injuries, aligning with the reported benefits of minimally invasive approaches. The incidence of other complications, such as periprosthetic fractures and dislocations, was not significantly different between the groups. Periprosthetic femoral fractures during surgery occurred at rates consistent with the literature (1.9%–10.3%) (24), highlighting the importance of cautious surgical technique, particularly for female patients with Dorr C-type proximal femurs or a low cortical thickness index, who might be more susceptible during the initial learning phase of the OCM approach (25).

Additionally, only one instance of dislocation was noted in the PLA group a month post-surgery, underscoring the efficacy of routine joint capsule suturing during surgery—a practice supported by existing literature to reduce dislocation risks (26). Moreover, variations in leg length were minimal and clinically acceptable, all within 10 millimeters (27). Long-term follow-up is essential to monitor for both common and rare, serious complications such as femoral arterial pseudoaneurysm (28), ensuring comprehensive assessment and management of postoperative outcomes.

Despite providing preliminary comparisons between the OCM and PLA methods in elderly patients, this study has limitations. First, the sample size is relatively small and the study is single-centered, which may limit the generalizability of the results. Secondly, the follow-up period is limited, primarily focusing on short-term clinical outcomes, with long-term effects yet to be verified. Future research should consider expanding the sample size and extending the follow-up period to more comprehensively assess the long-term effects of both surgical methods in a broader elderly population. Although a prospective randomized study indicated no significant advantages of the OCM method over the traditional approach in terms of mid-term clinical and functional outcomes (19), achieving better functional recovery in the short term is particularly important for elderly patients, and future studies should further explore the specific impacts of different surgical methods on short-term and long-term recovery in elderly patients.

In summary, the unique aspect of this study is its detailed comparison of the OCM and PLA surgical methods in patients aged 75 and older, especially in terms of promoting early functional recovery and reducing postoperative pain. The findings indicate that the OCM method has clear advantages over the PLA method in terms of surgical invasiveness, postoperative pain control, and early functional recovery. This discovery provides important references for clinical choice of surgical methods, particularly when considering the impact of postoperative recovery speed on the overall rehabilitation process for elderly patients.

## 5 Conclusions

Our findings demonstrate that the OCM approach provides significant short-term benefits for elderly patients undergoing bipolar hemiarthroplasty for femoral neck fractures, including faster recovery and reduced pain, without increasing complication rates compared to the PLA method. Despite its strengths, this study's limited follow-up and focus on an older demographic underscore the need for further research with broader and more diverse populations to validate and expand on these results. Nonetheless, the OCM approach emerges as a promising option for enhancing early functional outcomes in geriatric orthopedic care.

## Data Availability

The raw data supporting the conclusions of this article will be made available by the authors, without undue reservation.
